# Draft Genome Sequence of a Canine Uropathogenic Escherichia coli Strain Isolated in New Zealand

**DOI:** 10.1128/MRA.01665-18

**Published:** 2019-03-07

**Authors:** Vuong V. H. Le, Ian Bruce, Patrick J. Biggs, Jasna Rakonjac

**Affiliations:** a School of Fundamental Sciences, Massey University, Palmerston North, New Zealand; b New Zealand Veterinary Pathology Ltd., Palmerston North, New Zealand; c ^*m*^Epilab, Infectious Disease Research Centre, School of Veterinary Science, Massey University, Palmerston North, New Zealand; University of Rochester School of Medicine and Dentistry

## Abstract

Escherichia coli P50 is a canine uropathogenic isolate sampled in the Wellington region of New Zealand. We report the draft genome sequence of this isolate, which contains characteristic virulence genes for urinary tract infections and is predicted to be capable of causing human infections.

## ANNOUNCEMENT

Urinary tract infections (UTIs) affect about 150 million people each year, and uropathogenic Escherichia coli (UPEC) is the major causative agent (1). It was shown that UPEC isolates obtained from dogs with cystitis are able to invade human bladder epithelial cells and cause cytotoxicity, which emphasizes the zoonotic risk of canine UPEC isolates (2). In this report, we present the draft genome sequence of the isolate UPEC P50, which was isolated from a dog (12-year-old bull terrier) within a routine UTI diagnosis. This strain was isolated by culturing urine on MacConkey/sheep blood agar and identified as E. coli based on colony morphology on chromogenic UTI agar and using a Microbact 12A biochemical identification strip (Oxoid).

The genomic DNA was extracted from the overnight culture in 2xYT medium using the UltraClean microbial DNA isolation kit (Qiagen). The DNA sample was then submitted to the Massey Genome Service (Massey University, Palmerston North, New Zealand) for whole-genome shotgun sequencing using Illumina TruSeq Nano DNA library preparation and 2 × 300-base paired-end (PE) v3 sequencing on the Illumina MiSeq platform. The sequencing run generated 1,469,340 paired-end reads. The raw reads were trimmed to a quality cutoff value of Q30, and the short-length reads (<25 bases by default) were removed using SolexaQA^++^ v3.1.7.1 (3). After quality trimming, 1,303,860 paired-end reads with an average length of 200 bases per read were used for genome assembly, equivalent to 50× coverage for the draft genome. *De novo* assembly was performed with SPAdes v3.13.0 in the --careful mode (4). The gaps in the contigs were filled using GapFiller v1.10 with default parameters (5). Any contigs having a high identity with the phiX (ΦX174) sequence or Homo sapiens sequences were removed. The P50 draft genome was then annotated using the NCBI Prokaryotic Genome Annotation Pipeline (6). The genome assembly metrics were obtained using QUAST v5.0.1 with default parameters (7). Overall, the UPEC P50 draft genome is 5,155,240 bp long with 5,247 genes, and the GC content is 50.41%. There are 246 contigs with an *N*_50_ value of 314,239 bp, and the largest contig has 651,995 bp.

*In silico* analysis of the draft genome with SerotypeFinder server v2.0 (8), VirulenceFinder server v2.0 (9), and PathogenFinder server v1.1 (10) with default settings indicated that UPEC P50 has the O2:H1 serotype, possesses at least 10 virulence genes, including *cnf1*, *ireA*, *iroN*, *iss*, *mchB*, *mchC*, *mchF*, *mcmA*, *pic*, and *vat*, and has a high probability (*P* = 0.936) of causing human infections.

The UPEC P50 strain can be used as a model organism for studying the pathogenesis of UTIs and the genetics of biofilm formation and as a target for developing antibiotic therapies for treating UTIs and antibiotic-coated urinary catheters. The draft genome sequence of UPEC P50 would facilitate any precise genetic manipulation to support such types of research.

### Data availability.

The genome sequence of the UPEC P50 strain has been deposited at GenBank under the accession no. RQKE00000000. The raw data have been deposited at the SRA under the accession no. PRJNA506591.
